# Editorial Note: Reoviruses hijack the SMARCB1-MYC transcriptional regulation complex to activate autophagy for persistent viral infection in leafhopper vector

**DOI:** 10.1371/journal.ppat.1014318

**Published:** 2026-06-12

**Authors:** The PLOS Pathogens Editors

After publication of this article [1], concerns were raised regarding results presented in Fig 1D and S1 File.

Specifically:

- The Fig 1D RDV^–^ panels in [1] appear similar to the Fig 1D RSMV– panels in [2,3].
- In S1 File in [1], some of the underlying blots within a figure with a similar molecular weight range appear to show different blot characteristics, suggesting the proteins were detected on different gels and blots.

The corresponding author stated that the Fig 1D RDV^–^ panels in [1] are correct and the Fig 1D RSMV– panels in [2,3] are incorrect, and they provided the original image data underlying Fig 1D in [1] (S1 Underlying Data). The *PLOS Pathogens* Editors consider this concern resolved.

Regarding the blot data, the corresponding author stated that for each figure panel, all target proteins were detected from the same biological samples and replicates. They further stated that because different antibodies were required for different proteins, each protein was probed on a separate blot, and that the GAPDH and H3 reference control proteins were probed on different blots.

The *PLOS Pathogens* Editors issue this Editorial Note to inform readers of the above information and to relay the data provided.

## Supporting information

S1 Underlying Data Image data underlying Fig 1D. (TIF)
