# Supplementary figures and images for: Editorial Note: Reoviruses hijack the SMARCB1-MYC transcriptional regulation complex to activate autophagy for persistent viral infection in leafhopper vector
Source: PLoS Pathog. 2026 Jun 12;22(6):e1014318. doi: 10.1371/journal.ppat.1014318 (PMC13262889; doi:10.1371/journal.ppat.1014318)

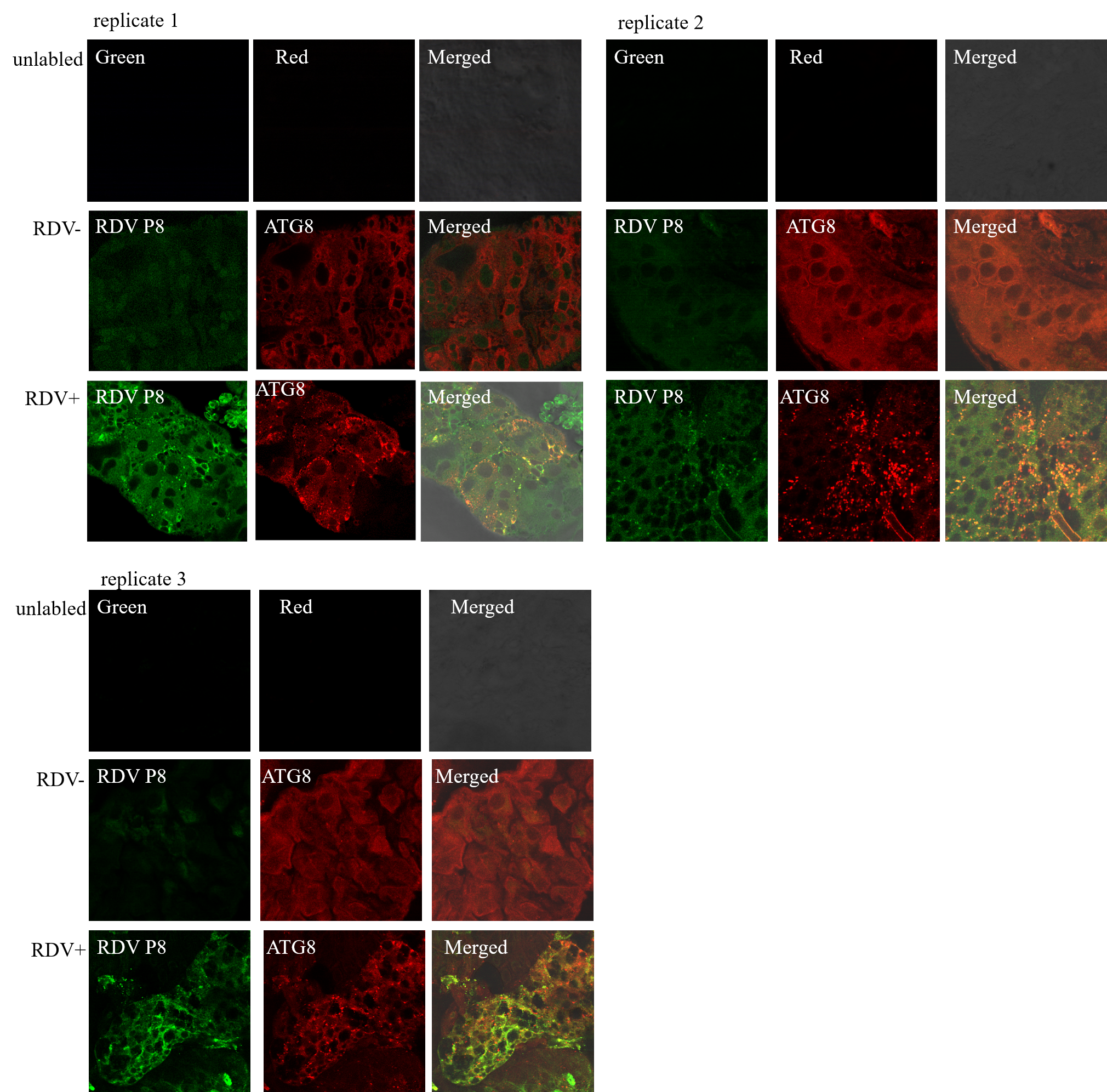

Supplement: S1 Underlying Data — (TIF) [file ppat.1014318.s001.tif]
